# Radiation induces progenitor cell death, microglia activation, and blood-brain barrier damage in the juvenile rat cerebellum

**DOI:** 10.1038/srep46181

**Published:** 2017-04-06

**Authors:** Kai Zhou, Martina Boström, C. Joakim Ek, Tao Li, Cuicui Xie, Yiran Xu, Yanyan Sun, Klas Blomgren, Changlian Zhu

**Affiliations:** 1 Center for Brain Repair and Rehabilitation, Institute of Neuroscience and Physiology, University of Gothenburg, Gothenburg, Sweden; 2 Karolinska Institutet, Department of Women’s and Children’s Health, Stockholm, Sweden; 3 Department of Oncology, Institute of Clinical Sciences, University of Gothenburg, Gothenburg, Sweden; 4 Perinatal Center, Institute of Neuroscience and Physiology, University of Gothenburg, Gothenburg, Sweden; 5 Department of Pediatrics, Zhengzhou Children’s Hospital, Zhengzhou, China; 6 Henan Key Laboratory of Child Brain Injury, Henan International Joint Laboratory of Child Brain Injury, Third Affiliated Hospital of Zhengzhou University, Zhengzhou 450052, China; 7 Department of Pediatric Oncology, Karolinska University Hospital, Stockholm, Sweden

## Abstract

Posterior fossa tumors are the most common childhood intracranial tumors, and radiotherapy is one of the most effective treatments. However, irradiation induces long-term adverse effects that can have significant negative impacts on the patient’s quality of life. The purpose of this study was to characterize irradiation-induced cellular and molecular changes in the cerebellum. We found that irradiation-induced cell death occurred mainly in the external germinal layer (EGL) of the juvenile rat cerebellum. The number of proliferating cells in the EGL decreased, and 82.9% of them died within 24 h after irradiation. Furthermore, irradiation induced oxidative stress, microglia accumulation, and inflammation in the cerebellum. Interestingly, blood-brain barrier damage and blood flow reduction was considerably more pronounced in the cerebellum compared to other brain regions. The cerebellar volume decreased by 39% and the migration of proliferating cells to the internal granule layer decreased by 87.5% at 16 weeks after irradiation. In the light of recent studies demonstrating that the cerebellum is important not only for motor functions, but also for cognition, and since treatment of posterior fossa tumors in children typically results in debilitating cognitive deficits, this differential susceptibility of the cerebellum to irradiation should be taken into consideration for future protective strategies.

Tumors of the posterior fossa account for up to 60% of all childhood intracranial tumors^1^. Most of the different types of posterior fossa tumors occur in childhood, including ependymomas, medulloblastomas, and pilocytic astrocytomas. Thirty percent of ependymomas are diagnosed in children under 3 years of age, most often located in the posterior fossa, and medulloblastomas and pilocytic astrocytomas account for 15–30% and 40% of all pediatric brain tumors, respectively^2^. Radiotherapy is one of the most effective treatments for brain tumors, but it is also a well-known risk factor for central nervous system injury^3^. Ionizing radiation induces oxidative stress, inflammation, stem/progenitor cell death, and microvascular injury^4^,^5^,^6^,^7^, all of which can lead to progressive cognitive decline and other behavioral abnormalities at long-term follow up^8^,^9^,^10^. These delayed and long-lasting adverse side effects of radiotherapy have serious impacts on the patient’s quality of life, and are recognized as important measurements of therapeutic outcomes of childhood brain tumors.

The mammalian cerebellum is located in the posterior cranial fossa and is critical for motor coordination and non-motor functions such as higher cognitive functions and emotional processing^11^,^12^,^13^,^14^. Irradiation of the cerebellum has multiple effects on its development, including reduction of its overall size, foliar/lobular malformations, and long-lasting blood-brain barrier (BBB) damage in the cerebrum and cerebellum of young mice^7^. A better understanding of irradiation-induced cerebellar pathology is needed to develop novel strategies to prevent the long-lasting side effects of radiotherapy. The purpose of this study was to define both short-term and long-term pathological changes in the cerebellum and to investigate possible mechanisms after cranial irradiation in juvenile rats.

## Results

### Irradiation-induced cell death was detected mainly in the EGL

**Figure 1:**
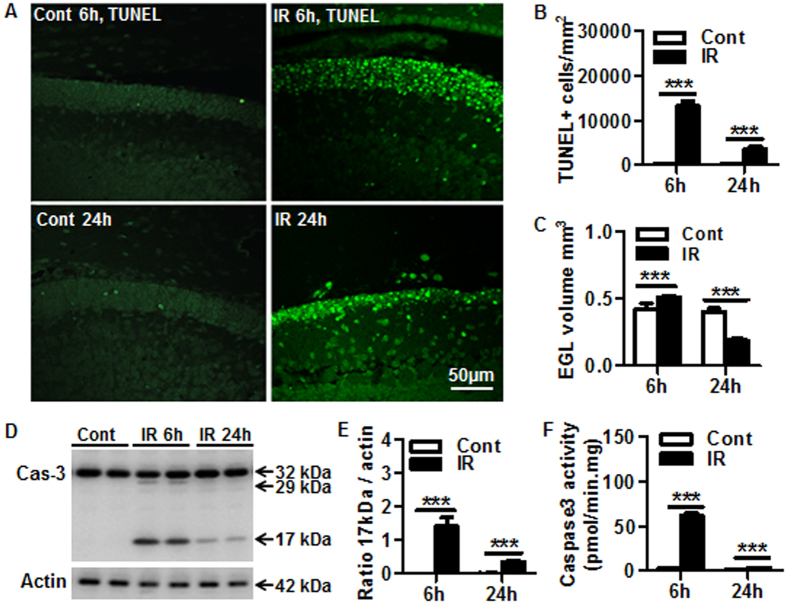
Irradiation-induced external germinal layer cell death and caspase-3 activity. (**A**) Representative TUNEL staining in the EGL of the cerebellum. (**B**) Quantification of TUNEL-labeled cells (n = 6/group). (**C**) The EGL volume increased at 6 h and decreased at 24 h after irradiation (IR) (n = 6/group). (**D**) Representative caspase-3 western blotting. (**E**) Quantification of the 17 kDa cleavage product (n = 5/group), normalized to actin. (**F**) Caspase-3 activity increased at 6 h and 24 h after irradiation compared to the controls (n = 9–10/group). EGL: external germinal layer. **p < 0.01, ***p < 0.001.

Cell death in the cerebellum was detected by TUNEL (Fig. 1A), labeling mainly cells located in the external germinal layer (EGL) in the irradiated brains (Fig. 1A, right panels). The total number of TUNEL-labeled cells was increased significantly at both 6 h and 24 h after irradiation compared with non-irradiated controls, with the greatest increase observed at the earlier time point (Fig. 1B). Based on H&E stained sections, the volume of the EGL increased 20.8% at 6 h after irradiation followed by a 52.9% decrease at 24 h after irradiation compared to controls (Fig. 1C). Caspase-3 activation in the cerebellum was determined by immunoblotting (Fig. 1D), and quantification of the 17 kDa cleavage product of caspase-3 showed a more prominent activation (cleavage) at 6 h compared to 24 h after irradiation (Fig. 1E). The immunoblotting results were further confirmed by an enzymatic assay, using Ac-DEVD-AMC as substrate (Fig. 1F).

### Cell proliferation and cell death of newborn cells in the cerebellum after irradiation

**Figure 2:**
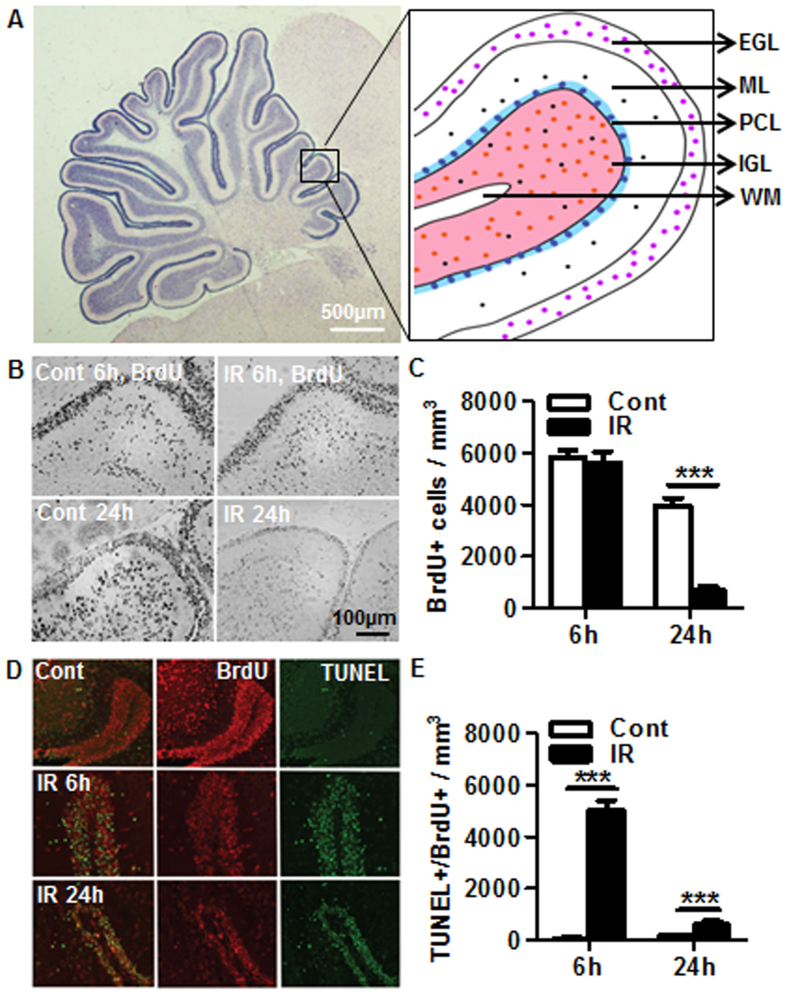
Irradiation-induced newborn cell death. (**A**) Representative folia of the cellular layers in the juvenile cerebellum. (**B**) Representative BrdU staining in the EGL of the cerebellum. (**C**) Quantification of BrdU-labeled cells showed no difference at 6 h, but the numbers were reduced by 82.9% at 24 h after irradiation compared to the controls. (**D**) Representative BrdU and TUNEL double labeling in the EGL of the cerebellum. (**E**) Confocal quantitative analysis of BrdU and TUNEL double-positive cells. n = 4 for control and n = 6 for irradiated. EGL: external germinal layer; ML: molecular layer; PCL: Purkinje cell layer; IGL: internal granule layer; WM: white matter. ***p < 0.001.

Cell proliferation in the cerebellum of the juvenile brain occurs mainly in the cerebellar EGL^15^ (Fig. 2A), which was measured by BrdU injection followed by BrdU immunostaining (Fig. 2B). The number of BrdU-labeled cells in the EGL was not different between control and irradiated animals at 6 h after irradiation but was reduced by 82.9% at 24 h after irradiation compared with non-irradiated controls (Fig. 2C). Cell death of these newborn cells was visualized by BrdU and TUNEL double-labeling (Fig. 2D), and quantification performed by confocal microscopy showed very few double-labeled cells in control brains, while most of the BrdU-labeled cells were TUNEL-positive at both 6 h (82.2%) and 24 h (91.7%) after irradiation (Fig. 2E).

### Microglia activation and inflammation in the cerebellum following irradiation

**Figure 3:**
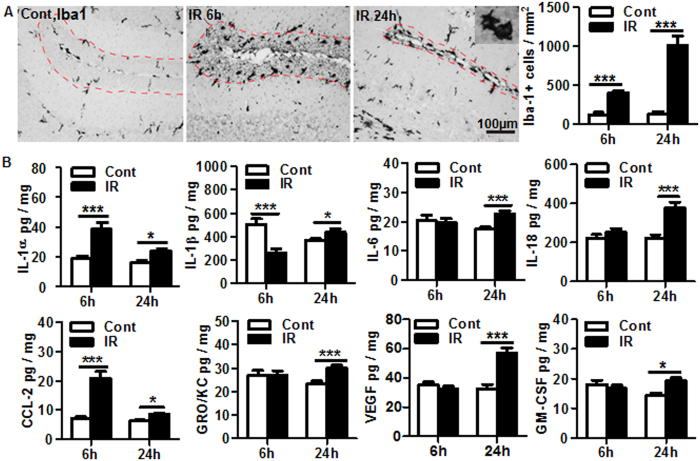
Irradiation-induced microglia proliferation and inflammation. (**A**) Representative Iba1 immunostaining in the EGL of cerebellum. The bar graph shows the quantification of Iba1-positive cells in the EGL and the density was increased dramatically after irradiation (n = 6/group). (**B**) Luminex assay of cytokines/chemokines in the cerebellum. IL-1α and CCL-2 increased significantly at both 6 h and 24 h after irradiation. IL-1β, IL-6, IL-18, GRO/KC, VEGF, and GM-CSF increased significantly at 24 h after irradiation (n = 9 or 10/group). EGL: external germinal layer. *p < 0.05, ***p < 0.001.

Ionized calcium-binding adapter molecule 1 (Iba1)-labeled cells (representing both non-activated and activated microglia) are evenly distributed throughout the EGL of the cerebellum. The density of microglia in the EGL was increased by 2.3 fold at 6 h (*p* = 0.00017) and by 6.77 fold at 24 h after irradiation (*p* = 0.00059), and most of the Iba1-positive cells had a bushy or amoeboid morphology (Fig. 3A). To measure the inflammatory response after irradiation, a panel of cytokines (IL-1α, IL-1β, IL-6, IL-18), chemokines (CCL-2, GRO/KC), and growth factors (VEGF, GM-CSF) were quantified in cerebellar lysates. IL-1α and CCL-2 increased by 2–2.8 fold at 6 h and remained significantly higher than controls at 24 h after irradiation (Fig. 3B). The expression of IL-1β was repressed at 6 hours post irradiation, followed by a slight increase at 24 hours post irradiation. IL-6, IL-18, GRO/KC, VEGF, and GM-CSF were all increased significantly at 24 h after irradiation compared to the control group (Fig. 3B).

### Oxidative stress in the cerebellum after irradiation

**Figure 4:**
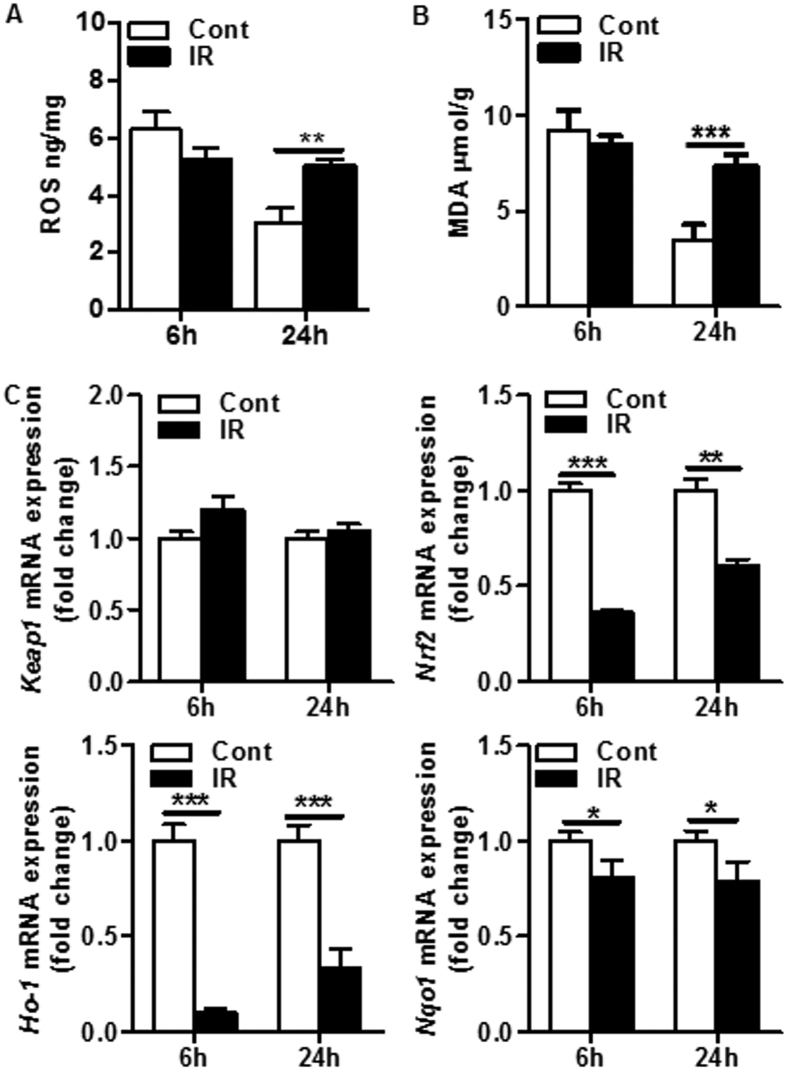
Irradiation-induced oxidative stress in the cerebellum. (**A**) Reactive oxygen species (ROS) in cerebellar homogenates increased significantly at 24 h after irradiation compared to controls (n = 9 or 10/group). (**B**) Malondialdehyde (MDA) in cerebellar homogenates also increased significantly at 24 h after irradiation compared to controls (n = 9 or 10/group). (**C**) mRNA expression of Nrf2-Keap1 signaling pathway-related genes at 6 h and 24 h after irradiation. *Nrf2, Ho-1*, and *Nqo1* mRNA decreased significantly at both 6 h and 24 h after IR, but there was no significant change in *Keap1* mRNA expression at any of the time points (n = 9 or 10/group). *p < 0.05, **p < 0.01, ***p < 0.001.

The production of free radicals was measured in the cerebellum at 6 h and 24 h after irradiation. Neither reactive oxygen species (ROS) (Fig. 4A) nor malondialdehyde (MDA) (Fig. 4B) were significantly different between controls and irradiated at 6 h. However, at the 24 h time point both ROS and MDA were significantly increased in irradiated mice compared to controls (Fig. 4A,B). We further measured gene expression of the Keap1-Nrf2 redox signal pathway^16^. The *Keap1* mRNA level remained unchanged after irradiation, but *Nrf2, Ho1*, and *Nqo1* mRNA decreased significantly both at 6 h (64%, 90%, 19%, respectively) and at 24 h (40%, 67%, 21%, respectively) after irradiation compared to controls (Fig. 4C).

### Blood-brain barrier and blood flow alterations in the cerebellum after irradiation

**Figure 5:**
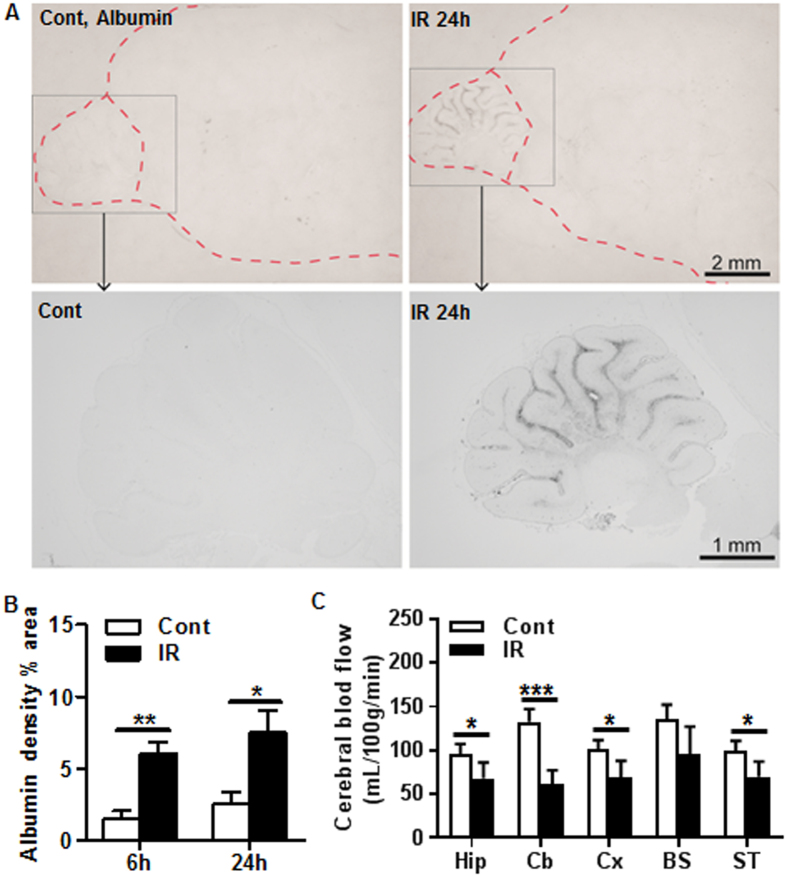
Irradiation increased blood-brain barrier (BBB) permeability and decreased blood flow in the cerebellum. (**A**) Representative albumin immunostaining in the cerebellum for control and irradiated animals at 24 hours post irradiation (top panel). (**B**) Albumin density in the cerebellum increased significantly at 6 h and 24 h after irradiation (n = 8/group). (**C**) Blood flow was decreased significantly in all the measured brain regions except in the brain stem at 6 h after irradiation. The reduction was most pronounced in the cerebellum (n = 9/group). Hippocampus: Hip; Cerebellum: Cb; Cortex: Cx; Brain stem: BS; Striatum/thalamus: ST. *p < 0.05, **p < 0.01.

BBB permeability was determined by albumin immunoreactivity in the extravascular tissue. Increased albumin staining was observed in the cerebellum of irradiated brains, but neither in control brains nor in any other brain regions after irradiation (Fig. 5A). The albumin positive staining increased significantly in the cerebellum as early as 6 h and persisted up to 24 h after irradiation (Fig. 5B). Cerebral blood flow (CBF) was assessed by the iodoantipyrine method in different brain regions and CBF significantly decreased at 6 h after irradiation in all measured brain regions, although not significantly so in the brain stem. The decrease in CBF was most pronounced in the cerebellum (53.7% reduction, *p* = 0.0003) compared to the cortex (31.4% reduction, *p* = 0.0213), hippocampus (29.1% reduction, *p* = 0.0414), brain stem (29% reduction, *p* = 0.0619), and the striatum/thalamus (29.9% reduction, *p* = 0.0394) (Fig. 5C).

### Mitochondrial fusion and fission in the cerebellum after irradiation

**Figure 6:**
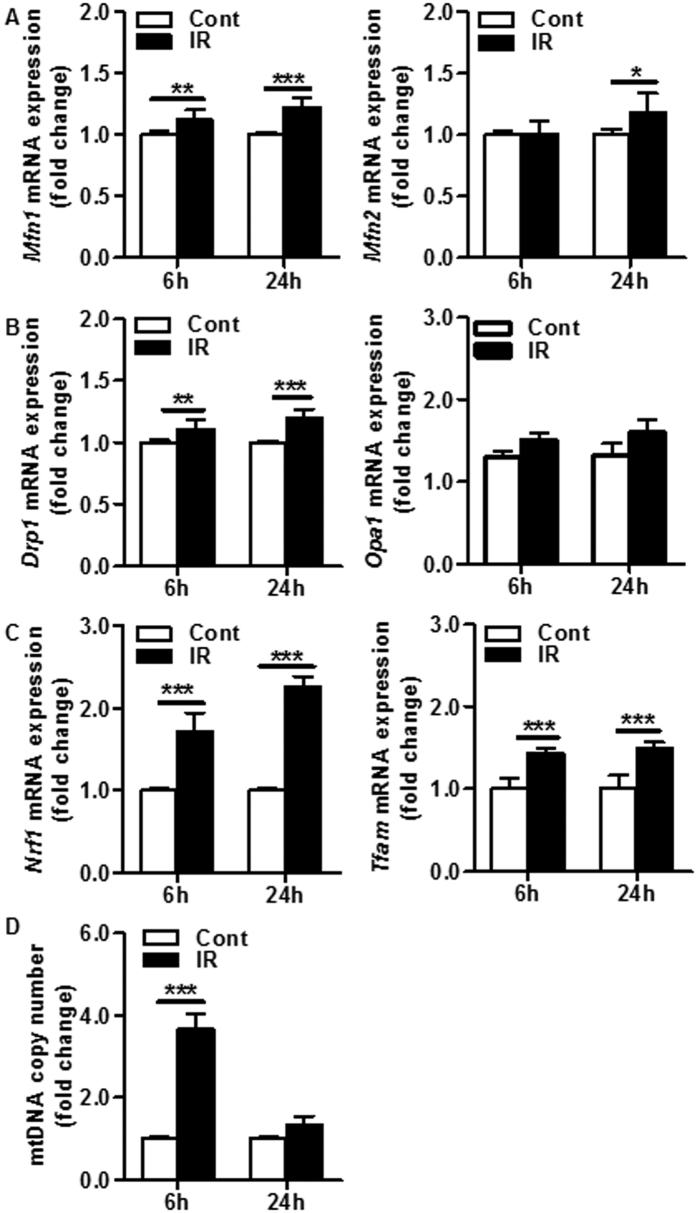
Impact of irradiation on mitochondrial fission, fusion, and biogenesis in the cerebellum. (**A**) The mRNA expression of the mitochondrial fusion genes *Mfn1* and *Mfn2* increased after irradiation. (**B**) The mRNA expression of the mitochondrial fission genes *Drp1* and *Opa1* increased after irradiation, but only the increase in *Drp1* mRNA was statistically significant. (**C**) The mRNA expression of the mitochondrial biogenesis genes *Nrf1* and *Tfam* increased dramatically after irradiation. (**D**) The mtDNA copy number increased 3.6 fold at 6 h after irradiation but was similar to the controls at 24 h after irradiation (n = 9 or 10/group). *p < 0.05, **p < 0.01, ***p < 0.001.

The dynamics of mitochondrial fusion and fission play critical roles in removing damaged mitochondrial DNA (mtDNA) in order to maintain respiratory function. Mitochondria fusion proteins (MFNs) are large transmembrane GTPases that bind and hydrolyze guanosine triphosphate to produce energy, where the two isoforms, Mfn1 and Mfn2 are located at the outer mitochondrial membrane^17^,^18^. The mRNA expression of *Mfn1* increased already at 6 hours post irradiation and at 24 hours post irradiation the mRNA expression of both isoforms was still elevated (Fig. 6A). Mitochondrial fission is regulated by dynamin-related protein 1 (Drp1) and optic atrophy 1 (Opa1)^19^. Although there was no significant difference in the expression of *Opa1*, the expression of *Drp1* gene was significantly increased after irradiation at both investigated time points (Fig. 6B). In addition, the mRNA levels of the mitochondrial biogenesis-related genes *Nrf1* and *Tfam* increased significantly at both 6 h and 24 h after irradiation (Fig. 6C). Finally, the mtDNA content, as indicated by mtDNA copy number, increased 3.6 fold at 6 h after irradiation but was similar to controls at 24 h after irradiation (Fig. 6D).

### Long-term effects of irradiation in the cerebellum

**Figure 7:**
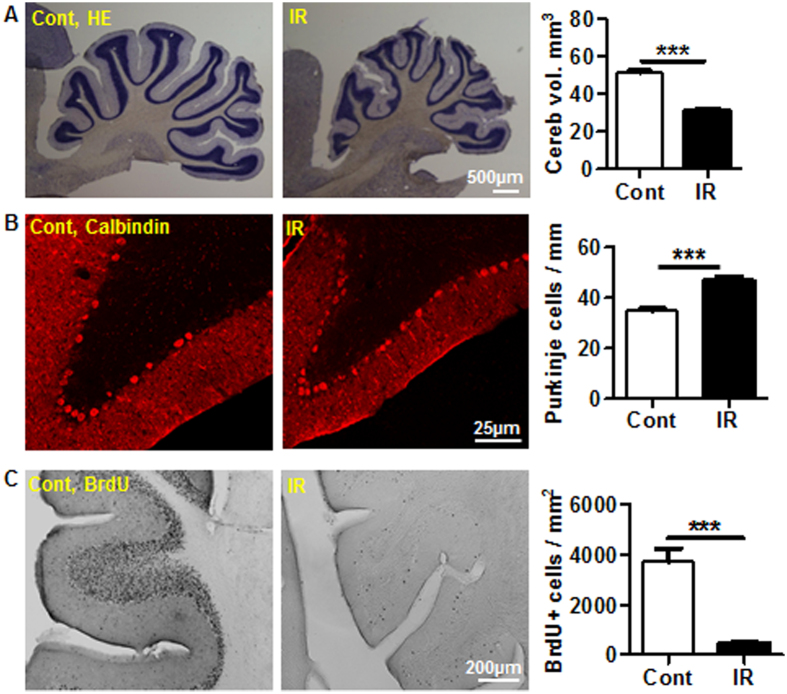
Long-term effects of irradiation on the cerebellum. (**A**) Representative hematoxylin and eosin staining. The quantification of the cerebellar volume showed a 39% decrease at 16 weeks after irradiation (n = 11 for the control group, n = 17 for the irradiation group). (**B**) Representative calbindin staining for Purkinje cells at 16 weeks after irradiation. The number of calbindin-labeled cells increased by 35% at 16 weeks after irradiation (n = 8 for the control group, n = 12 for the irradiated group). (**C**) Representative BrdU staining in the cerebellar lobe in controls at 16 weeks after irradiation. The number of BrdU-positive cells in the internal granule layer decreased by 87.5% 16 weeks after irradiation (n = 5/group). ***p < 0.001.

In the current study, long-term irradiation-induced effects in the cerebellum were evaluated 16 weeks after irradiation. Volume measurements revealed a 39% reduction of the total cerebellar volume after irradiation (Fig. 7A), with similar reductions observed in both gray and white matter. Calbindin staining was used to quantify the total number of Purkinje cells in the cerebellum, which revealed 35% greater density of Purkinje cells in the irradiated animals (Fig. 7B). The migration of BrdU-labeled cells to the internal granule layer (IGL) was decreased by 87.5% in irradiated brains (Fig. 7C).

**Figure 8:**
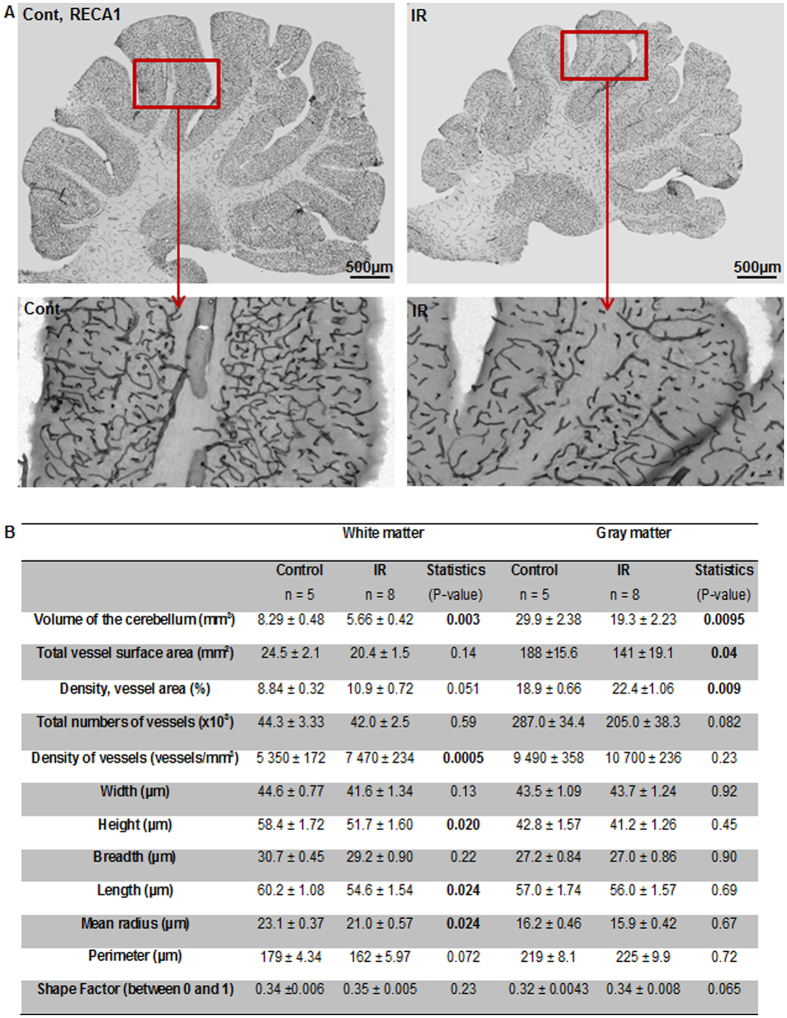
The long-term effect of irradiation on microvessels. (**A**) Representative RECA-1 staining of endothelial cells in the cerebellum (top panel), and a higher magnification of cerebellar lobe V (bottom panel). (**B**) Quantitative stereological analysis of microvessels in both the white and gray matter. Microvessel height, length, and mean radius in the white matter and the total vessel surface area in the gray matter decreased significantly at 16 weeks after irradiation (n = 5 for the control group, n = 8 for the irradiated group).

With the use of RECA-1 immunostaining, different microvessel parameters in the gray and white matter of the cerebellum were visualized and quantified (Fig. 8A). The total vessel surface area was reduced after irradiation, however only significantly so in the gray matter. In addition, no significant difference for the total number of vessels was found in neither the white nor gray matter, although there was a tendency towards a decrease in the irradiated gray matter (p = 0.082). Since the irradiated cerebellum displayed reduced growth, we also calculated the densities. This revealed an increased density of the vessel area in the gray matter, and a trend towards an increase in the white matter (p = 0.051). Furthermore, the density of vessel numbers was significantly increased in the white matter. Structural microvessel parameters, such as width, height, breadth, length, mean radius, and perimeter were measured, and revealed a significant decrease of height, length, and mean radius in the white matter, but not in the gray matter (Fig. 8B).

## Discussion

Irradiation has several deleterious effects on the brain, which depend on both age and radiation dose^20^. The irradiation dose used in this study (6 Gy) is equivalent to 12 Gy when delivered in repeated daily 2 Gy fractions (as in a clinical setting), according to the linear-quadratic model^21^ using an α/β ratio of 3 for late effects in normal brain tissue. This represents a clinically relevant, low to moderate radiation dose. The neurogenic regions, such as the subventricular zone and the dentate gyrus in the hippocampus, are particularly susceptible to irradiation and have therefore attracted much attention during recent years^5^,^22^. Although the cerebellum also exhibits prolonged postnatal neurogenesis, it is often ignored in studies of the effect of irradiation because the germinal layer disappears quickly during cerebellar development^23^. In the current study, we found that cell proliferation is much more prominent in the EGL than other layers of the juvenile cerebellum and that irradiation induces extensive death of proliferating cells, blood flow reduction, and BBB damage, as well as persistent and substantial cerebellar lack of growth or atrophy. All of these observed findings are likely to be correlated to the long-term adverse effects of cerebral radiotherapy in children.

It has been demonstrated that proliferating cells are particularly sensitive to irradiation^4^. Irradiation-induced stem and progenitor cell death in the developing brain is age-dependent^20^ and has been shown to be associated with cognitive impairments. Prevention of stem cell death and cerebellar atrophy might therefore improve behavioral functions^10^. Two germinal zones exist in the cerebellum, the ventricular zone and the external germinal zone. The Purkinje cells are derived from the ventricular zone, a process that finishes before birth. However, proliferation of stem and progenitor cells in the EGL continues and peaks at 15 days and disappears at 24 days after birth in rodents (it disappears between 12 months and 18 months after birth in humans)^15^. Because of the early completed development of the cerebellum, the potentially negative effects of radiation on the cerebellum have unfortunately been neglected in previous research. In this study, we found significant cell death in the EGL of the cerebellum after irradiation as indicated by TUNEL labeling. The EGL volume increased at 6 h but shrunk at 24 h after irradiation, which might be related to irradiation-induced death of proliferating cells and edema at 6 h and the removal of most of the dead cells and the disappearance of edema at 24 h after irradiation^24^. Irradiation induces DNA damage directly or indirectly through the generation of free radicals, resulting in apoptosis and the loss of proliferating cells^22^. In the current study, irradiation induced widespread apoptotic cell death in the EGL of the juvenile cerebellum, and this was accompanied by caspase-3 activation, which indicates that irradiation-induced cell death in the cerebellum is at least partly due to caspase-3–dependent apoptosis. Furthermore, the dead cells were mostly newborn (proliferating) cells, as indicated by BrdU/TUNEL double labeling. These results are consistent with the findings in other neurogenic brain regions after irradiation, especially in the hippocampus^4^,^25^.

Irradiation-induced oxidative stress is a result of disturbance between production of free radicals and antioxidant defense mechanisms and is characterized by an increase in ROS and MDA as well as a decrease in antioxidants^5^. The brain contains high levels of polyunsaturated fatty acids, making it more susceptible to oxidative damage, and ROS scavengers have been demonstrated to be effective in ameliorating the neuronal injury that is induced by radiation exposure^26^. In this study, we found that irradiation induced oxidative stress as indicated by increased levels of ROS and MDA. This is in line with the findings in a study of the adult mouse cerebellum that used MDA and 8-OHdG as oxidative stress markers^27^. We also investigated the mRNA level of the genes encoding the Nrf2/Keap1 complex, which is a very important factor for ameliorating oxidative stress. Interestingly, we found that *Nrf2* mRNA decreased significantly after irradiation, which would likely disrupt the Nrf2/Keap1 association and decrease the cells’ capacity to respond to oxidative stress. We also found that two other important oxidative stress regulators, heme oxygenase-1 (HO-1) and quinone reductase (NQO1), were downregulated after irradiation. This implies that irradiation-induced oxidative stress is at least partly due to decreased production of antioxidants.

Microglia are resident mononuclear phagocytes that maintain brain microenvironment homeostasis and provide immune defense. They become activated after insults to the brain, and such activation is characterized by rounding of the cell body, retraction of cell processes, proliferation^28^,^29^, and increased production of ROS, cytokines, and chemokines that mediate neuroinflammation^6^,^30^. Microglia activation plays an important role in phagocytosis of dead cells, but persistent microglia activation contributes to chronic inflammation and increased susceptibility to subsequent injuries, as demonstrated by the use of anti-inflammatory treatments to prevent irradiation-induced cognitive impairment^31^,^32^,^33^. In the current study, irradiation induced acute microglia proliferation and increased the production of pro-inflammatory cytokines and chemokines in the cerebellum, an effect which is similar to other neurogenic regions^29^,^34^. These results provide a rationale for the use of anti-inflammatory interventions to prevent or ameliorate radiation-induced juvenile cerebellar injury.

Radiation-induced endothelial cell death is of crucial importance for the development of brain injury^35^,^36^ since this may lead to structural changes in blood vessels, decreased blood vessel density and length, and increased vessel permeability^7^,^37^. In the current study we observed that cranial irradiation to the juvenile rat resulted in a lack of growth of the cerebellum, both in the gray and white matter. Moreover, irradiation also affected the vascular status in the cerebellum by for example reducing total vessel surface area at 16 weeks post irradiation. However, when the vascular parameters were normalized to the volume of the cerebellum we observed increased densities of the vessel area and numbers of vessels. These results indicate a sustained effect of irradiation on the vasculature, or that the surrounding tissue suffers an even greater volume loss and growth arrest than the vascular bed. This is in stark contrast to a previous study where we only observed an initial increased vessel density in the hippocampus (one week after irradiation) after a single dose of 8 Gy towards the brain of P14 mice^38^. This was followed by a normalization with time (at 7 weeks up to one year after irradiation), indicating that the vasculature in the hippocampus (compared to the cerebellum) adapted to the altered needs of the surrounding tissue^38^. Similar results were observed in the corpus callosum where both the vessel surface and numbers of vessels were reduced 4 months post irradiation but no difference were observed when calculating vascular densities, hence arguing against the theory of the white matter vasculature being more sensitive to irradiation compared to the gray matter^39^. However, in the current study of cerebellum we observed that vascular parameters such as height, length and mean radius were only reduced in the white matter of irradiated brains, indicating that in some aspects the cerebellar white matter is in fact more sensitive than cerebellar gray matter. Taken together, this indicates that the growing cerebellum is more sensitive to irradiation that other brain regions.

Endothelial cell death precedes acute BBB disruption and takes several weeks to recover^40^, a prolonged recovery that might be related to persistent microglia activation and inflammation in the irradiated brain. Studies have also identified endothelial cells as a critical component of the neural stem cell niche and shown that they are involved in the regulation of stem and progenitor cell proliferation and differentiation^41^,^42^. Irradiation disrupts the neurovascular niche both in the adult and juvenile developing brain^41^,^43^, and might influence stem and progenitor cell proliferation and differentiation, as well as injury and repair. In the juvenile rat brain, we found that BBB disruption after irradiation varies among brain regions and that the cerebellum is the most sensitive region to irradiation in this respect. This finding is in line with another study demonstrating BBB disruption 1 week and 4 weeks post irradiation in the cerebellum of both juvenile and adult mouse brains^7^. However, BBB integrity in the cerebrum was only disrupted after 1 week and in the adult brain and after 4 weeks in the juvenile brain indicating that the cerebellar BBB is more sensitive to cranial irradiation. We did not find any disruption of the BBB in the cerebrum of young rats in this study. Also in young mice we did not find any BBB disruption in the cerebrum, as judged by infiltration of monocyte-derived macrophages^29^. Furthermore, in the current study irradiation-induced blood flow reduction was most pronounced in the cerebellum out of all the different brain regions. Vascular endothelial growth factor (VEGF) is a growth factor that stimulates vasculogenesis and angiogenesis and is known to mediate increased vascular permeability and edema^44^. It has been suggested that VEGF might modulate immune responses in the central nervous system by opening the BBB. Overexpression of VEGF in the brain contributes to BBB breakdown^45^, and the increased VEGF expression observed in the current study might be related to irradiation-induced endothelial cell death and tissue hypoxia, leading to persistent BBB breakdown or act as compensatory rescuing mechanism to repair the BBB damage.

The main target of ionizing radiation damage is believed to be the DNA in the nucleus; however, there is evidence suggesting that mtDNA is even more susceptible to irradiation/oxidative damage because of the fact that it is not protected by histones^46^. Therefore, mitochondria are likely to be a major target of ionizing radiation in addition to the genomic DNA in the nucleus^47^. Mitochondria undergo permanent fusion and fission to balance energy demand and nutrient supply, and they also undergo fusion to remove damaged mtDNA and to maintain the respiratory function^48^,^49^. We found that the expression of fusion genes increased after irradiation, probably related to self-preservation and an attempt to remove the damaged mtDNA. However, we observed that mitochondrial fission gene expression also increased after irradiation, likely related to maintaining the high-level balance of fusion and fission in the mitochondria. Mitochondrial biogenesis is crucial for functional recovery after injury, and both *in vitro* and *in vivo* studies have shown that this process is increased after irradiation^50^,^51^,^52^. These results are in accordance with our findings of increased mitochondrial biogenesis-related genes and mtDNA copy number after irradiation.

In summary, we characterized radiation-induced pathological changes in the juvenile rat cerebellum. We found that BBB damage and reduced blood flow was greater in the cerebellum compared to other brain regions after irradiation. Furthermore, radiation-induced apoptotic cell death in the EGL contributed to the reduction of granule cells in the IGL and to cerebellar atrophy. These findings shed light on the mechanisms occurring during cerebellar development and might have important implications for radiation risk assessment. Importantly, this knowledge could be used to develop novel approaches to reduce long-term radiotherapy-induced side effects in the treatment of pediatric brain tumors.

## Materials and Methods

### Animals and irradiation procedure

Male Wistar rats were purchased from Charles River Laboratories (Germany). On postnatal day 11 (P11), the rats were anesthetized with a 50 mg/kg intraperitoneal injection of tribromoethanol (Avertin, Sigma-Aldrich), placed in a prone position on a styrofoam bed. The whole brain was irradiated with a symmetrical 2 × 2 cm radiation field and the head was covered with a 1 cm tissue-equivalent bolus material. A linear accelerator (Varian Clinac 600CD, Radiation Oncology Systems, San Diego) with 4 MV nominal photon energy and a dose rate of 2.3 Gy/min was used to irradiate mice with a single absorbed dose of 6 Gy. Sham-irradiated controls were anesthetized but not subjected to irradiation. All experiments were approved by the Gothenburg Committee of the Swedish Animal Welfare Agency (application no. 202-2012) and all the experimental methods were performed in accordance with the guidelines for animal experiments of Gothenburg University.

### BrdU administration

The thymidine analog 5-bromo-2-deoxyuridine (BrdU, Roche) was freshly prepared prior to use (5 mg/ml in 0.9% saline). In order to label proliferating cells before irradiation at P11, BrdU was injected intraperitoneally at a dose of 50 mg/kg on P9 and P10. For the long-term study, BrdU injections were also given on P12 and P13.

### Immunohistochemistry staining

At 6 h or 24 h after irradiation, rats were deeply anesthetized with 50 mg/ml pentobarbital and perfused intracardially with PBS. Brains were removed, and the left hemisphere was immersed in 5% formaldehyde in 0.1 M PBS (Histofix, Histolab) for 24 h at 4 °C, followed by dehydration with graded ethanol and xylene. The brains were paraffin embedded and cut into 5-μm thick sagittal sections. Every 50^th^ section from the hippocampal level was stained. After antigen recovery and blocking, the sections were incubated with rabbit anti-Iba1 (1:500, WAKO) and monoclonal rat anti-BrdU (1:200, clone: BU1/75, Oxford Biotechnology Ltd.) primary antibodies at room temperature (RT) for 60 min. After washing and incubating with the appropriate biotinylated secondary antibodies (1:500, Vector Laboratories) at RT for 60 min, visualization was performed using Vectastain ABC Elite with 3,3′-diaminobenzidine. For TUNEL labelling, sections were incubated with 3% bovine serum albumin in 0.1 M Tris-HCl (pH 7.5) for 30 min followed by 50 μl TUNEL reaction mixture for 60 min at 37 °C in a humidified chamber. After washing, sections were mounted using Vectashield mounting medium. For double labeling of TUNEL and BrdU, after staining with TUNEL, the sections were incubated with rat anti-BrdU antibody at RT for 60 min and then incubated with Alexa 555 donkey anti-rat IgG (H + L) (1:1000) for 60 min, washed and mounted using Vectashield mounting medium with DAPI.

For the long-term study, animals were sacrificed at 16 weeks after irradiation. Half hemisphere was transferred into 30% sucrose in 0.1 M PBS after fixing in Histofix for 24 h. The brains were cut sagittally into 30-μm thick sections on a sliding microtome (Leica SM 2000R), and then stored in tissue cryoprotectant solution at −20 °C. BrdU labelling was performed on free-floating sections. DNA denaturation was achieved by incubating the sections in 2 mol/L HCl for 30 min at 37 °C, rinsing in 0.1 mol/L borate buffer (pH 8.5) for 10 min and rinsing in Tris-buffered saline (pH 7.5). Sections were incubated with 3% donkey serum for 30 min and then incubated with rat anti-BrdU antibody (1:200; AbD Serotec) for 16 h at 4 °C. After rinsing with TBS, sections were incubated for 2 h at RT with biotinylated donkey anti-mouse antibody (1:1000; Jackson ImmunoResearch Laboratories). After rinsing, sections were incubated with avidin−peroxidase for 60 min (ABC-kit; Vector Laboratories) and then with detection solution (26.5 mg/mL diaminobenzidine, 0.01% H_2_O_2_, 0.04% NiCl). For calbindin and endothelial cell staining, sections were blocked with 3% donkey serum after antigen retrieval and then incubated with goat anti-calbindin (1:200, Santa Cruz) or rat anti-endothelial cell antibody (1:200, RECA-1, Abcam) in blocking solution for 16 h at 4 °C. After rinsing, the sections were incubated for 2 h with Alexa 555 donkey anti-goat IgG (H + L) (1:1000) or biotinylated donkey anti-rat secondary antibody (1:500). The biotinylated antibody was visualized according to diaminobenzidine chromogenic procedure described above.

### Cell quantification and volume measurement

BrdU-, Iba1-, and TUNEL-labeled cells were counted in the EGL of the cerebellum. Counting was performed on same-layer sections on lobules 2–4 for BrdU and TUNEL. Iba1 was counted in the EGL on all the lobules^53^. Purkinje cells were counted along the IGL on lobules 2–4. Regional volumes were calculated according to the Cavalieri principle using the formula described previously^30^.

### Assessment of the blood-brain barrier

The integrity of the BBB to endogenous albumin was assessed immunocytochemically at 6 and 24 h after irradiation. Sections were incubated with a sheep anti-albumin antibody (1:1000, Bethyl Laboratories) overnight followed by biotinylated horse anti-sheep antibody (1:250; Vector) for 2 h and avidin-peroxide complex (ABC kit, Vector) for 1 h. Sections were then developed with nickel-enhanced diaminobenzidine. For quantification, photomicrographs were obtained of the cerebellum using standardized light and camera settings. Images were analyzed using Image J software (1.48 v). The images were converted to 8-bit gray scale, the cerebellum was outlined, and the threshold function was used to differentiate areas with positive albumin immunoreactivity. Using uniform threshold settings, the fraction of albumin-positive area (%) in the cerebellum was measured for each animal, which was used as the measurement of BBB integrity. Other areas of the brain remained effectively negative.

### Luminex assay

The right hemisphere cerebellum was collected at 6 and 24 h after irradiation and homogenized by sonication in 1 ml ice-cold 50 mM Tris-HCl (pH 7.3) solution containing 5 mM EDTA. Homogenate samples were centrifuged at 10000 × *g* for 10 min at 4 °C and protein concentration in the supernatant determined using the bicinchoninic acid protein assay kit (Thermo). The rat cytokine/chemokine magnetic bead panel from Millipore was performed on a Bio-Plex 200 system with the Bio-Plex Manager software 6.0 (Bio-Rad) following the protocol provided by the kit manufacturer.

### Western blotting

Homogenate samples with a volume of 65 μL were mixed with 25 μL NuPAGE LDS 4× sample buffer and 10 μL reducing agent (ThermoFisher Scientific) and heated at 70 °C for 10 min. Individual samples were run on 4–12% NuPAGE Bis-Tris gels (Novex) and transferred to reinforced nitrocellulose membranes. After blocking with 30 mM Tris-HCl (pH 7.5), 100 mM NaCl and 0.1% Tween 20 containing 5% fat-free milk powder for 60 min at RT, the membranes were incubated with the rabbit anti-actin (1:200; Sigma) or rabbit anti-caspase3 (1:1000; Santa Cruz) primary antibodies at RT for 60 min. After washing, membranes were incubated with a peroxidase-labeled goat anti-rabbit secondary antibody (1:2000, Vector Laboratories) for 30 min at RT. Immunoreactive species were visualized using the Super Signal West Dura substrate (ThermoFisher Scientific) and an LAS 3000 cooled CCD camera (Fujifilm).

### Caspase-3 activity and ROS/MDA assay

The caspase-3 activity assay was performed as described previously^54^, and the MDA assay (Cayman Chemical) was performed according to the protocol provided by the manufacturer. Briefly, 100 μl homogenate samples or standards, 100 μl SDS, and 4 ml color reagent were mixed in 15 ml vials. The vials were placed in vigorously boiling water for 1 h and then immediately removed and placed in an ice bath for 10 min to stop the reaction, followed by 30 min incubation at RT. A total volume of 150 μl was added to a clear plate and the absorbance was read at 530–540 nm. ROS were measured with the Rat ROS ELISA kit (NeoScientific) following the manufacturer’s protocols. Briefly, 50 μl homogenate sample or standard was added to the corresponding well and then incubated 1 h at RT. The wells were emptied and washed three times before adding 100 μl of conjugate per well and incubated for 1 h at 37 °C. This was followed by washing five times, incubating with 50 μl substrate for 15 min and then adding 50 μl stop solution to each well. Finally, the optical density of each well was measured at 450 nm.

### Regional cerebral blood flow

Regional cerebral blood flow was measured at 6 h after irradiation by the iodoantipyrine method as previously described^55^. Measurements were made in the hippocampus, cortex, striatum/thalamus, cerebellum, and brain stem. Briefly, 10 μCi (100 μL) 4-Iodo [N-methyl-^14^C] antipyrine (American Radiolabeled Chemicals) was injected subcutaneously. At 60 seconds after injection, rat pups were decapitated, blood was collected, and brain regions for measurements were immediately dissected out and weighed. All tissues were dissolved in Solvable^TM^ (Perkin-Elmer) followed by the addition of 4.5 mL Ultima Gold^TM^ (Perkin Elmer). The radioactivity in the samples (dpm/g tissue) was then measured by liquid scintillation counting (Packard Instrument). The result is expressed as mL/100 g/min.

### Mitochondrial DNA copy number

Total cerebellar DNA was isolated using the DNeasy Blood & Tissue kit (Qiagen). The amount of mitochondrial DNA (mtDNA) relative to nuclear genomic DNA was determined by quantitative real-time PCR. Genomic DNA was measured with the *Hprt* gene using the primers 5′-AGGCTGGACTCTAAATCATAATC-3′ (sense) and 5′-CTGCTGTCT GTATTGGTGTAT-3′ (anti-sense), and mtDNA was measured with the *ND4* gene using the primers 5′-AACTCAATAGGAACACTCAACTT-3′ (sense) and 5′-AGATGGACTCCGTAT AATGGTA-3′ (anti-sense). Relative mtDNA levels were calculated based on the threshold cycle (Ct) as 2^−Δ(ΔCt)^.

### Quantitative real-time PCR

Total RNA was isolated with the RNeasy mini kit (Qiagen) according to the manufacturer’s instructions. Concentration and purity of all RNA samples were determined using a Nanodrop spectrophotometer (Nanodrop Technologies) and integrity of the RNA was measured using the Experion^TM^ RNA StdSens Analysis kit (Bio-Rad) on an Automated Electrophoresis Station machine (Bio-Rad). One microgram of total RNA was reverse transcribed using the QuantiTect^®^ Reverse Transcription kit (Qiagen). Quantitative real time PCR was performed using the Light Cycler 480 instrument (Roche Diagnostics) and the SYBR Green technique according to the manufacturer’s instructions. The primers used in the qPCR reactions were designed by Beacon Designer software (PREMIER Biosoft) and are listed in Supplementary Table 1. Relative expression levels of mRNAs were calculated using the method of geometric averaging of multiple internal control genes.

### Analysis of cerebellar vascular morphology

With the help of Stereo Investigator’s virtual tissue module, multiple images were acquired and stitched together in order to create one single image of the RECA-1 vessel stain in the whole cerebellum using a Leica light microscope at 10x magnification. All vessel morphology analyses where performed using the integrated morphometry analysis module in the Metamorph Offline software (Metamorph^®^, Molecular Devices, Inc.). Each image was manually inspected, and a threshold was set to define the positive RECA-1 staining against the background. All analyses were measured in pixels and later recalculated into μm (1 pixel = 0.732 μm or 0.536 μm^2^). In this study, the following parameters were analyzed: ① total vessel surface area; ② total number of vessels; ③ vessel width; ④ vessel height; ⑤ vessel breadth; ⑥ vessel length; ⑦ mean radius; ⑧ vessel perimeter; and ⑨ shape factor: a value between 0 and 1 describing how closely the vessel approximates a circle, where a value near 0 indicated a flattened object and a value of 1 indicated a perfect circle. The shape factor was calculated using the following equation: 4πA/P^2^, where P = perimeter and A = area.

### Statistical analysis

All data are expressed as means ± SEM. Student’s *t*-test was used when comparing two groups, and *p*-values less than 0.05 were considered to be statistically significant.

## Additional Information

**How to cite this article**: Zhou, K. *et al*. Radiation induces progenitor cell death, microglia activation, and blood-brain barrier damage in the juvenile rat cerebellum. *Sci. Rep.* **7**, 46181; doi: 10.1038/srep46181 (2017).

**Publisher's note:** Springer Nature remains neutral with regard to jurisdictional claims in published maps and institutional affiliations.

## Supplementary Material

Supplementary Dataset
