# Supplementary material for: Radiation induces progenitor cell death, microglia activation, and blood-brain barrier damage in the juvenile rat cerebellum
Source: Sci Rep. 2017 Apr 6;7:46181. doi: 10.1038/srep46181 (PMC5382769; doi:10.1038/srep46181)
Supplement: Supplementary Dataset [file srep46181-s1.doc]

**Radiation induces progenitor cell death, microglia activation, and blood brain barrier damage in the juvenile rat cerebellum**

Kai Zhou^1,2^, Martina Boström^1, 3^, Joakim Ek^4^, Tao Li ^1,5^, Cuicui Xie^1,2^, Yiran Xu^1,6^, Yanyan Sun^1,6^, Klas Blomgren^2,7^, Changlian Zhu^1,6*^

1. Center for Brain Repair and Rehabilitation, Institute of Neuroscience and Physiology, University of Gothenburg, Gothenburg 40530, Sweden
2. Karolinska Institutet, Department of Women’s and Children’s Health, Stockholm, Sweden
3. Department of Oncology, Institute of Clinical Sciences, University of Gothenburg, Sweden
4. Perinatal Center, Institute of Neuroscience and Physiology, University of Gothenburg, Sweden
5. Department of Pediatrics, Zhengzhou Children’s Hospital, Zhengzhou, China
6. Henan Key Laboratory of Child Brain Injury, Henan International Joint Laboratory of Child Brain Injury, Third Affiliated Hospital of Zhengzhou University, Zhengzhou 450052, China
7. Department of Pediatric Oncology, Karolinska University Hospital, Stockholm, Sweden

**Supplementary table 1.  qPCR primer sequences**

| Gene | Primer sequence | Type | Product size(bp) |
| --- | --- | --- | --- |
| *Tfam* | Sense: 5’- GAGTTCTGCCGTTTGCTTA -3’  Antisense: 5’- TTCAGTCTGTCTACACCATCA -3’ | Mitochondrial biogenesis | 97 |
| *Nrf1* | Sense: 5’- CCAAGCATTACGGACCATAG -3’  Antisense: 5’- GTCTGTACTACTGTCTGTGATG -3’ | Mitochondrial biogenesis | 150 |
| *Drp1* | Sense: 5’- TGCTCAGTATCAGTCTCTTCT-3’  Antisense: 5’- GCCGTTCCTTCAATAGTGTTA-3’ | Mitochondrial fission | 120 |
| *Opa1* | Sense: 5'- GACGCAGCCATCTACTTC-3'  Antisense: 5'- TCATCATTAACCTTCAGCATCTT-3’ | Mitochondrial fission | 188 |
| *Mfn1* | Sense: 5’- GCTGGACATCTGGATTGATAA-3’  Antisense: 5’- GCCGCTCATTCACCTTAT-3’ | Mitochondrial fusion | 119 |
| *Mfn2* | Sense: 5’- AACGCTGAACTTCTTACTGT -3’  Antisense: 5’- TCTCTAACTCTACCTGTCCTAC -3’ | Mitochondrial fusion | 136 |
| *Nrf2* | Sense: 5’- GGATGGGAAACCTTACTCTC -3’  Antisense: 5’- TATCTGGCTTCTTGCTCTTG -3’ | Keap1-NRF2 pathway | 92 |
| *Keap1* | Sense: 5’- GGAAAGAAGGAACAGGTGAG -3’  Antisense: 5’- TGGGAAAGTTACAGGGCTAT -3’ | Keap1-NRF2 pathway | 134 |
| *Nqo1* | Sense: 5’- GCAGAGAGGACATCATTCAA -3’  Antisense: 5’- TTCATAGCATAGAGGTCAGATTC -3’ | Keap1-NRF2 pathway | 101 |
| *Ho-1* | Sense: 5’- GTCTCTCTGGAATGGAAGGA -3’  Antisense: 5’- CTCTACCGACCACAGTTCT-3’ | Keap1-NRF2 pathway | 125 |
| *Gapdh* | Sense: 5’- GCTGCCTTCTCTTGTGAC-3’  Antisense: 5’- CTTGACTGTGCCGTTGAA-3’ | House keeping gene | 123 |
| *B2m* | Sense: 5’- ATGGCTACTTCTGCTTTGTTA-3’  Antisense: 5’- AACCACTTCACTTCACTCTG-3’ | House keeping gene | 103 |
